# Special Issue on “Smart Homes”: Editors’ Notes

**DOI:** 10.3390/s19040836

**Published:** 2019-02-18

**Authors:** Alessandro Freddi, Sauro Longhi, Andrea Monteriù

**Affiliations:** Department of Information Engineering, Università Politecnica delle Marche, Via Brecce Bianche, 60131 Ancona, Italy; a.freddi@univpm.it (A.F.); s.longhi@univpm.it (S.L.)

**Keywords:** intelligent household, Internet of Things, interoperability, smart monitoring, smart living, safety, security, energy management, home automation, smart sensors, wireless sensor networks, machine learning, home appliances and devices

## Abstract

In this editorial, we provide an overview of the content of the Special Issue on “Smart Homes”. The aim of this Special Issue is to provide a comprehensive collection of some of the current state-of-the-art technologies in the context of smart homes, together with new advanced theoretical and technological solutions that enable smart technology diffusion into homes.

## 1. Special Issue Overview

Advances in Information Communication Technologies (ICTs) have made interoperability possible, such that everyday devices at home can be networked to give the inhabitants new and unexpected possibilities. In particular, the high penetration rate of the Internet of Things (IoT) paradigm in household environments allows introducing the new concept of the “smart home”, namely a residence equipped with innovative technological solutions and services to improve residents’ living and security. Smart Home Technologies (SHTs) comprise sensors, monitors, interfaces, appliances, and devices networked together to enable automation, as well as localized and remote control of the domestic environment. In this context, thanks to the latest sensor technologies and machine learning algorithms, the domestic technological environment is able to monitor the well-being and daily life activities of inhabitants, to learn their specific needs and habits, and to adapt itself to them, thus improving their overall quality of life. In addition, smart homes can intelligently manage the energy usage of appliances and all other aspects of the domestic environment, thus creating a more comfortable, energy-efficient space for their inhabitants. Smart home technologies have also a great impact on home automation, health, independent and assisted living, security, and many others. This Special Issue is devoted to new research efforts and results and developments and applications in the area of sensors and technologies for intelligent household.

## 2. Main Topics Addressed in the Published Papers

A total of 25 papers were submitted to this Special Issue. After the review phase, 13 papers were accepted. The focus of these papers is mainly on the following three topics: human monitoring, interoperability and usability, and energy management. In the following, a brief overview of each paper is proposed: the main challenges faced by each paper are highlighted, together with a minimal description of the proposed solutions.

### 2.1. Human Monitoring

Human monitoring plays an important role in smart homes, such as in assisted living scenarios or emergency detection. A fundamental problem in human monitoring is how to localize humans in indoor environments. In [1], a localization method was proposed for tracking humans’ position in indoor environments based on Passive Infrared (PIR) sensors. First, the grid-based accessibility map, which reflects human visiting preferences and the physical layout of the area, is built. Then, PIR sensors, deployed according to the grid-based accessibility map, provide an external rough position of the human. Finally, an A-star algorithm fuses the PIR sensor data and grid-based accessible map information to estimate the trajectory and reduce the errors. Experiments have been performed in a mock apartment testbed, and the method could prove especially useful in service robotics applications. The position of people within a smart home can then be used as the base for more complex services. For instance, the work in [2] proposed a smart terminal for remote “one-click” control of devices, driven by the position of the user estimated via fingerprint matching based on received signal strength analysis, together with pedestrian dead reckoning. The solution has been tested in a laboratory scenario. Human monitoring, in its deeper sense, aims to recognize important events, especially those that may hinder people’s safety. Fall detection is one of the most investigated events in assistive solutions. The authors in [3] proposed an accurate fall detection method investigating the depth frames of the human body using a single device in a top-view configuration, with the subjects located under the device inside a room. Features extracted from depth frames train a classifier based on a binary support vector machine learning algorithm. A fall is identified when the distance between the Kinect and the centroid associated with the person’s head becomes comparable with the floor distance. Comprehensive monitoring, however, requires using several sensor (a so-called sensor network) and inferring useful information from a noticeable amount of data. In order to exploit such information, however, powerful analytics is needed to convert raw sensor output into meaningful and accessible knowledge. In [4], a complete monitoring architecture was presented, which relies on a cloud-based approach. Behavioral explanatory models are introduced, with the purpose of quantitatively monitoring a given quantity of interest (e.g., toilet visits across time). Then, a tool that models the probability of the activation of given sensors throughout the day is presented: this enables a statistical hypothesis testing frame for detecting changes between two different time periods. Finally, a methodology to extract complex user patterns was presented, based on machine-learning techniques to infer meaningful information from sensor data, thus dealing with the inherent variability of human behaviors. The system has been deployed at several pilot sites and tested on real data. The authors of [5], instead, focused more on the smart sensing architecture, presenting an integrated sensor network to monitor the user and the environment in order to derive information about the user’s behavior and her/his health status. The proposed platform includes biomedical, wearable, and unobtrusive sensors for monitoring user’s physiological parameters and home automation sensors to obtain information about her/his environment. The sensor network stores the heterogeneous data both locally and remotely in the cloud, where machine learning algorithms and data mining strategies are used for user behavior identification, classification of user health conditions, classification of the smart home profile, and data analytics to implement services for the community. The proposed solution has been experimentally tested in a pilot study based on the development of both sensors and services for elderly users at home. Whenever monitoring is performed via cameras, the problem of “privacy” arises. The authors of [6] investigated the detection of embarrassing situations for social robots in smart homes using convolutional neural networks. The paper aimed to protect the sensitive information at the beginning of video data collection, thanks to a developed mechanism, which permits a social robot to detect embarrassing situations and convert privacy information to non-sensitiveinformation. This is done via an improved neural network structure and feature extraction algorithms based on You Only Look Once (YOLO). The algorithm has been trained with a dataset of six classes of situations in the smart home: taking a shower, sleeping (naked or half-naked), using the toilet, dressing (naked or half-naked), humans are in the smart home, but no privacy context is involved, and no person in the smart home. Tests were performed on different sets of images.

### 2.2. Interoperability and Usability

A smart home depends on the communication and cooperation among numerous devices, which integrate computation, networking, and physical processes and are able to monitor and control physical objects, providing an extremely efficient and economic means for improving the quality of life and the security of people. One challenge is to exploit low-cost heterogeneous sensor devices for data collection and to develop the specialized software that is able to analyze and correlate the different heterogeneous sources of information, by using the computation facilities commonly available in most of the houses. The main purpose of [7] is to demonstrate that it is possible to leverage any environment as a smart one by using basic, off-the-shelf technologies and applications. Three levels of rules are provided, and a solution is proposed that decouples the different levels of control rules, so as to maximize their effectiveness and to reduce as much as possible their maintenance and updating effort. The proposed infrastructure has been validated in a simplified scenario. Another challenge is that of ensuring proper functionalities of all the sensors installed within a smart home. In this regard, the authors of [8] presented a systematic literature review of the sensor failure detection and fault tolerance in ambient assisted living applications. The existing works were discussed, and the limitations and research gaps were highlighted. Software-level interoperability was instead faced by [9]. A solution to ease interoperability between heterogeneous devices belonging to the same system and also between heterogeneous Cyber Physical Systems (CPSs) was proposed. In particular, the paper described the design and implementation principles based on a component technology called COScore, which assists the extension of this kind of system by modifying its underlying software architecture through model abstractions. The approach was validated through an example scenario with different subsystems of a smart home solution. Interoperability must also be ensured at the interface level. In this regard, the integration of a spoken dialog system can provide a more convenient experience to the user. The authors in [10] proposed a framework that enables developers to design an intelligent dialog system effectively. The framework ontologically expresses the knowledge required for the task-oriented dialog system’s process and can build a dialog system by editing the dialog knowledge. In addition, the framework provides a module router that can indirectly run externally-developed modules. Finally, it enables a more intelligent conversation by providing a Hierarchical Argument Structure (HAS) to manage the various argument representations included in natural language sentences. With the proposed intelligent dialog system, even beginner dialog system developers can develop a high-level task-oriented dialog system.

### 2.3. Energy Management

Human monitoring, devices’ interoperability, and user interfaces are topics of primary importance for usability, safety, and comfort of people within a smart home. Another important aspect to consider, however, is that of energy management and energy conservation, which have an impact both on the comfort and economical sides. Appliance scheduling is widely accepted as an effective mechanism to manage domestic energy consumption. The authors of [11] proposed a smart home energy management system that reduced unnecessary energy consumption by integrating an automated switching off system with a load balancing and appliance scheduling algorithm. The load balancing scheme acts according to defined constraints, such that the cumulative energy consumption of the household is managed below the defined maximum threshold. The scheduling of appliances adheres to the Least Slack Time (LST) algorithm, while considering user comfort during scheduling at the same time. The performances of the proposed scheme were proven through computer simulation. At the same time, maintaining a balance between the energy consumption cost and users’ comfort satisfaction is a challenge. In [12], the main objective was to shift load profiles in home appliances, as well as cut down peak energy demands, by using a new constrained swarm intelligence-based residential consumer-centric Demand-Side Management (DSM) method, which takes into account predictable day-ahead Real-Time Pricing (RTP), Inclining Block Rates (IBR), and consumers’ comfort satisfaction. A constrained Particle Swarm Optimization (PSO)-based residential consumer-centric load scheduling method was proposed, which can be further implemented with edge computing. A new approach that is linked with the progress of technologies (smart metering, sensors, and actuators) and that allows detailed electricity consumption analytics was detailed in [13]. This method is able to forecast the residential electricity consumption down to the appliance level, using sensor-recorded data, for residential smart home complexes that utilize renewable energy sources as a part of their consumed electricity. In order to attain this goal, a mixed Artificial Neural Network (ANN) approach is employed, which exploits both Non-linear AutoRegressive with eXogenous input (NARX) ANNs and function FITting neural NETworks (FITNETs).
